# Construction of high-quality recombination maps with low-coverage genomic sequencing for joint linkage analysis in maize

**DOI:** 10.1186/s12915-015-0187-4

**Published:** 2015-09-21

**Authors:** Chunhui Li, Yongxiang Li, Peter J. Bradbury, Xun Wu, Yunsu Shi, Yanchun Song, Dengfeng Zhang, Eli Rodgers-Melnick, Edward S. Buckler, Zhiwu Zhang, Yu Li, Tianyu Wang

**Affiliations:** Institute of Crop Sciences, Chinese Academy of Agricultural Sciences, Beijing, China; Institute for Genomic Diversity, Cornell University, Ithaca, NY USA; USA Department of Agriculture-Agricultural Research Service, New York, USA; Department of Crop and Soil Sciences, Washington State University, Pullman, WA USA

**Keywords:** Recombination bin map, Sequencing, Joint linkage analysis, Nested association mapping population, Maize

## Abstract

**Background:**

A genome-wide association study (GWAS) is the foremost strategy used for finding genes that control human diseases and agriculturally important traits, but it often reports false positives. In contrast, its complementary method, linkage analysis, provides direct genetic confirmation, but with limited resolution. A joint approach, using multiple linkage populations, dramatically improves resolution and statistical power. For example, this approach has been used to confirm that many complex traits, such as flowering time controlling adaptation in maize, are controlled by multiple genes with small effects. In addition, genotyping by sequencing (GBS) at low coverage not only produces genotyping errors, but also results in large datasets, making the use of high-throughput sequencing technologies computationally inefficient or unfeasible.

**Results:**

In this study, we converted raw SNPs into effective recombination bins. The reduced bins not only retain the original information, but also correct sequencing errors from low-coverage genomic sequencing. To further increase the statistical power and resolution, we merged a new temperate maize nested association mapping (NAM) population derived in China (CN-NAM) with the existing maize NAM population developed in the US (US-NAM). Together, the two populations contain 36 families and 7,000 recombinant inbred lines (RILs). One million SNPs were generated for all the RILs with GBS at low coverage. We developed high-quality recombination maps for each NAM population to correct genotyping errors and improve the computational efficiency of the joint linkage analysis. The original one million SNPs were reduced to 4,932 and 5,296 recombination bins with average interval distances of 0.34 cM and 0.28 cM for CN-NAM and US-NAM, respectively. The quantitative trait locus (QTL) mapping for flowering time (days to tasseling) indicated that the high-density, recombination bin map improved resolution of QTL mapping by 50 % compared with that using a medium-density map. We also demonstrated that combining the CN-NAM and US-NAM populations improves the power to detect QTL by 50 % compared to single NAM population mapping. Among the QTLs mapped by joint usage of the US-NAM and CN-NAM maps, 25 % of the QTLs overlapped with known flowering-time genes in maize.

**Conclusion:**

This study provides directions and resources for the research community, especially maize researchers, for future studies using the recombination bin strategy for joint linkage analysis. Available resources include efficient usage of low-coverage genomic sequencing, detailed positions for genes controlling maize flowering, and recombination bin maps and flowering- time data for both CN and US NAMs. Maize researchers even have the opportunity to grow both CN and US NAM populations to study the traits of their interest, as the seeds of both NAM populations are available from the seed repository in China and the US.

**Electronic supplementary material:**

The online version of this article (doi:10.1186/s12915-015-0187-4) contains supplementary material, which is available to authorized users.

## Background

Maize exhibits extremely high levels of genetic diversity and phenotypic variation [1, 2]. Numerous single nucleotide polymorphisms (SNPs), small insertions-deletions, and large structural variations substantially contribute to this genetic diversity [3–5]. Abundant genetic variation gives rise to great challenges for uncovering the genetic basis of quantitative phenotypic variation.

Studies undertaken to understand the genetic architecture of complex traits and their variations in maize have generally been performed by linkage analysis and/or association mapping [6]. Linkage analysis based on a bi-parental population only detects two alleles and has poor mapping resolution. A genome-wide association study (GWAS) often identifies spurious associations due to population structure and genetic relatedness [7]. Joint linkage analysis, using multiple segregating populations, could overcome some of the inherent limitations associated with single population linkage analysis and genome-wide association mapping [8].

To provide an effective genomic resource for joint linkage analysis in maize, US researchers created a nested association mapping (NAM) population. About 5,000 recombinant inbred lines (RILs) were created by crossing 25 diverse inbred lines with a common parent, B73 [8]. US-NAM has been effectively used with genetic, genomic, and systems biology tools to dissect the genetic architecture of agronomic traits in maize [9–16]. In addition, analyses with US-NAM have been quite accurate at surveying the magnitude of effects across diverse germplasm [17].

One shortcoming of US-NAM, however, is its limited number of founder lines; a NAM population becomes more effective for GWAS when the allele is found in three or more founders [18]. Additionally, because only one reference line was used in US-NAM, the genetic and phenotypic biases introduced into the population are unknown.

To further improve our understanding of the genetic architecture of maize and improve the resolution of quantitative trait loci (QTLs), Chinese researchers independently developed a new NAM population (CN-NAM). About 2,000 RILs were created by crossing 11 diverse elite inbred lines with a common parent HUANGZAOSI (HZS). Parents of the CN-NAM population were selected from different heterotic groups that are widely used in Chinese maize breeding [19]. Combining the CN-NAM and US-NAM populations can provide a larger genomic resource for dissecting the genetic architecture of complex traits in maize. Furthermore, combining populations can enable higher power and higher resolution in genetic mapping through joint linkage analysis.

Despite the availability of this new mapping method and the larger mapping population, marker density remains an important limiting factor for identifying genes controlling quantitative traits. This limitation is especially pronounced for fine mapping of QTLs. New sequencing technologies have facilitated cost-effective high-throughput SNP genotyping for natural and artificial mapping populations. Genotyping by sequencing (GBS), using methylation-sensitive restriction enzymes to reduce genome complexity while retaining good genome coverage of lower copy regions, has proven to be a highly efficient method in different species [20–25]. Yet, GBS-obtained data could be as large as millions of SNPs, a density that makes the construction of a composite genetic map almost impossible and negates the potential benefits of conducting joint linkage analysis in NAM populations.

One strategy to address this issue is to construct a recombination bin map by identifying exact recombination breakpoints and dividing the chromosomal regions into small recombination bins. These small recombination bins can be regarded as an effective type of genetic marker [26]. This construction method of sequencing-based high-density genetic maps could substantially reduce the amount of time and effort required for QTL mapping. For example, this method has been used for constructing bin maps of single bi-parental populations and QTL mapping of complex agricultural traits in rice and sorghum [27–29]. Therefore, we expected that recombination bin maps would have the potential to improve the statistical power of joint linkage analysis and reduce the computing time of QTL mapping in NAM populations.

The objectives of this study were the following: (1) to construct high-resolution recombination bin maps of both the US-NAM and CN-NAM populations based on GBS data; (2) to evaluate the quality and accuracy of bin maps by joint linkage analysis of a flowering-related trait, days to tasseling; and (3) to conduct a combined analysis of different NAM populations.

## Results

### Identification of high-quality lines

All RILs of the 36 families that comprised the two NAM populations were sequenced with GBS, resulting in a total of one (0.95) million SNPs. After removing monomorphic and MAF < 0.05 SNPs across all families from the raw GBS data, 238,945 and 294,962 SNPs were obtained for CN-NAM with 11 families and US-NAM with 25 families, respectively. Based on these SNPs, we identified outlier lines of two NAM populations. After removing the outliers, we obtained 1,696 and 4,623 high-quality RILs in the CN-NAM and US-NAM populations, respectively. We used these data for the following analyses.

### Bin maps of individual families

**Fig. 1:**
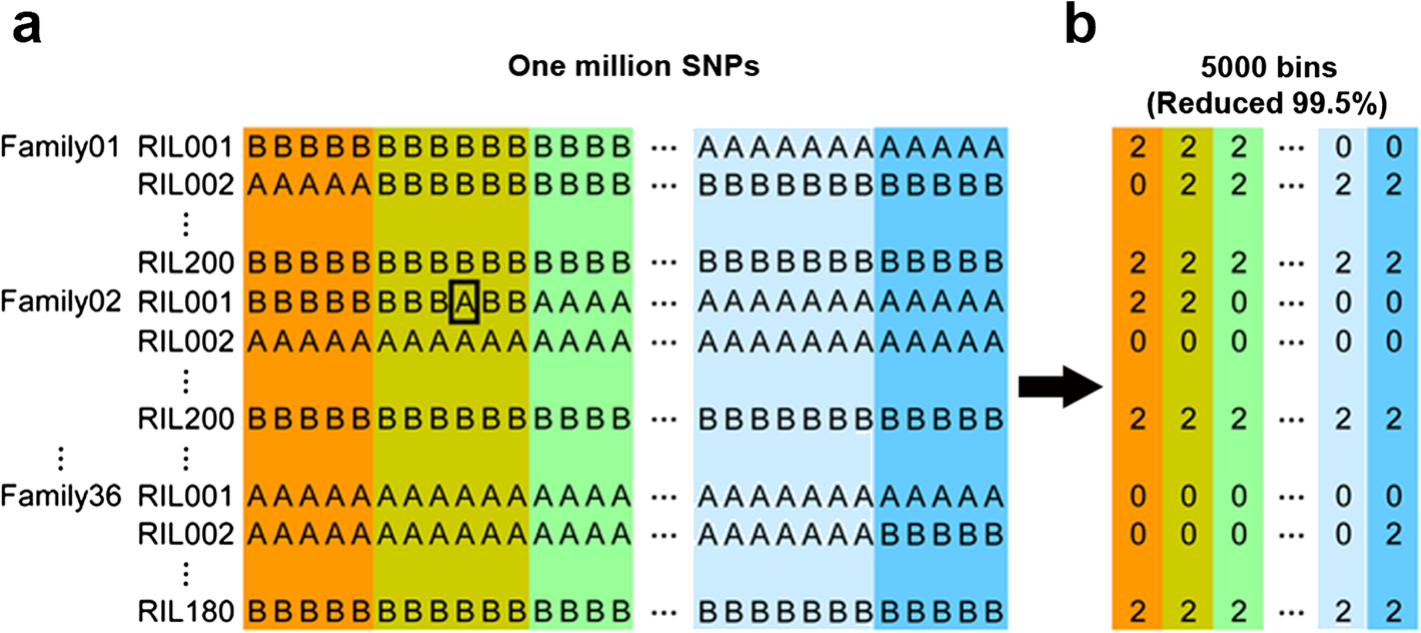
Construction of effective recombination bins from raw SNPs in NAM populations. **a** SNP genotypes of both NAM populations were obtained by genomic sequencing. **a** and **b** represent genotypes from common parents and genotypes from diverse parents, respectively. Genotypes indicated by a rectangular box were incorrect due to raw sequencing errors. **b** Bin map was constructed with sequencing SNPs by recombination breakpoint location. 0 and 2 represent genotypes from common parents and genotypes from diverse parents, respectively. A hidden Markov model was used to correct the error of genotype data

After removing low-quality SNPs, the number of high-quality SNPs ranged from 39,620 for the HUANGYESI3 family in CN-NAM to 109,104 for the CML228 family in US-NAM, with the corresponding mean SNP density ranging from about 1 SNP/63.1 kb to 1 SNP/22.9 kb, respectively. These SNPs were used to infer parental genotypes, and detect recombination breakpoints for all available RILs, as indicated by Fig. 1. The total number of breakpoints per individual family ranged from 3,552 for 160 RILs in the HUANGYESI3 family to 6,695 for 179 RILs in the CML228 family. The average number of breakpoints per individual RIL in each family ranged from 22.2 to 37.4 (Additional file 1: Table S1). After recombination breakpoint locations were determined, the genotype of each RIL was assigned.

**Fig. 2:**
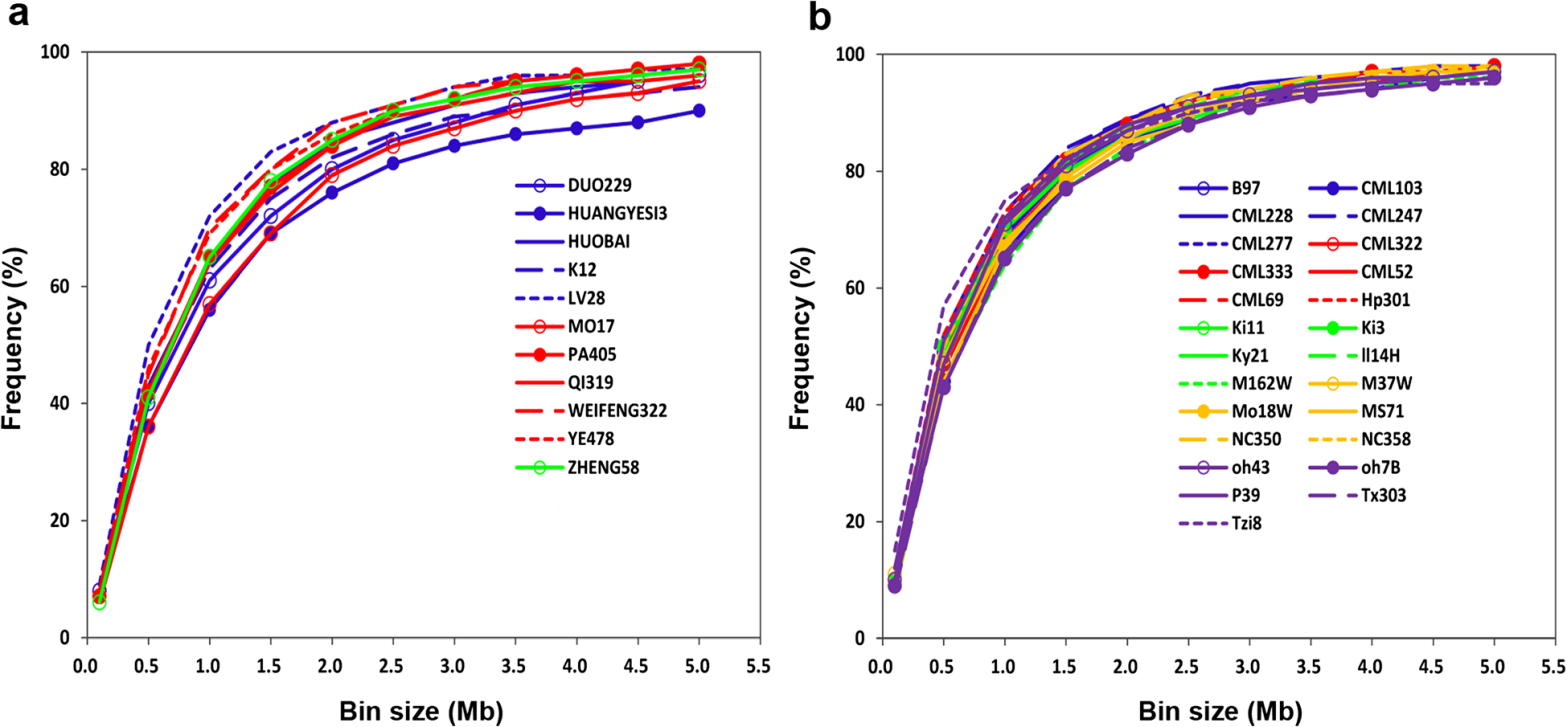
Cumulative distribution of recombination bin sizes within each family of **a** CN-NAM and **b** US-NAM

Bin maps were constructed for each family based on the identity with the parental genotypes. The number of recombination bins varied substantially among the 36 families. The total bin number per individual family ranged from 734 to 2,183. More than 50 % of the bins were less than 1,000 kb in physical length for all families in both CN-NAM and US-NAM (Fig. 2). The median physical length of bins within an individual family ranged from 402 kb for the Tzi8 family (US-NAM) to 852 kb for the HUANGYESI3 family (CN-NAM). Detailed bin information for the 36 families is provided in Additional file 2: Table S2.

Genetic linkage maps were constructed for each family using bin genotypes. The genetic length of individual family maps ranged from 1,178.58 cM for the HUANGYESI3 family to 2,035.58 cM for the CML228 family. The average genetic distance between adjacent bins ranged from 0.77 cM for the CML333 family to 1.61 cM for the HUANGYESI3 family.

### Bin maps of CN-NAM and US-NAM

Joint recombination bin maps were constructed using 5,435 and 5,692 bins (without missing data) from CN-NAM and US-NAM, respectively (Additional file 3: Figure S1). The physical length of the bins ranged from 5.0 kb to 5.6 Mb (with an average of 378 kb) and from 5.0 kb to 9.2 Mb (with an average of 327 kb) in CN-NAM and US-NAM, respectively. In total, 92 % of bins were less than 1 Mb in length in CN-NAM compared to 94 % in US-NAM. Subsequently, of the 5,435 CN-NAM bins, 2,706 with no segregation distortion were used to construct an initial framework genetic map. The remaining markers (bins) with segregation distortion were added to the framework map, and we ensured that no marker order or chromosome position conflicts were introduced. Finally, a total of 4,932 bins were selected to construct a composite genetic map of CN-NAM, resulting in a genetic distance of 1,700.44 cM in length, with an average genetic distance of about 0.34 cM between adjacent bins. Likewise, of the 5,692 US-NAM bins, 2,278 with no segregation distortion were used to construct a framework map. Eventually, 5,296 bins were selected for the composite genetic map of US-NAM, yielding a 1,456.68-cM genetic length, with an average genetic distance of about 0.28 cM between adjacent bins (Additional file 4: Table S3).

### Quality and accuracy of bin maps

The quality and accuracy of these maps for QTL mapping were evaluated by locating the known flowering-time genes for days to tasseling (DT). Detailed results can be found in Additional file 5: Table S4 and Additional file 6: Table S5.

DT, one of the most obvious measures of reproductive growth in maize, is thought to involve male and female flowering-related genes [30]. The broad-sense heritability for DT was 0.90 in CN-NAM and 0.89 in US-NAM. Therefore, we checked for the presence of known genes involved in the regulatory pathway of flowering time within the interval of DT-related QTLs in different genetic backgrounds (Additional file 7: Figure S2). For example, *ZMM4,* a maize MADS-box gene in the FUL1 family, promotes floral transition and inflorescence development in maize [31]. In this study, four QTLs detected under different genetic backgrounds overlapped the *ZMM4* gene, with a likelihood of odd (LOD) score ranging from 3 to 13, and explained 6.9 % to 34.9 % of the phenotypic variation. One of four QTLs had its LOD peak in bin 0231 in the M37W family, which encompassed a region of 780 kb in length and completely contained the *ZMM4* locus. Within the limits of single-family mapping resolution, the *ZMM4* overlapping DT QTLs found in different families is evidence that the individual family bin strategy can produce maps with high quality and accuracy.

Further evidence came from mapping the *ZCN8* gene. The flowering time *ZCN8* may function as the florigen and integrates signals from both photoperiod and autonomous pathways in maize [32]. We identified QTLs overlapping the *ZCN8* region in four families. Among the four QTLs, the largest effect was detected in the HUANGYESI3 family, explaining 37 % of the phenotypic variation. Within the QI319 family, two major QTLs were detected on chromosomes 8 and 10 and completely overlapped the cloned *ZCN8* and *ZmCCT* genes (Additional file 7: Figure S2).

**Fig. 3:**
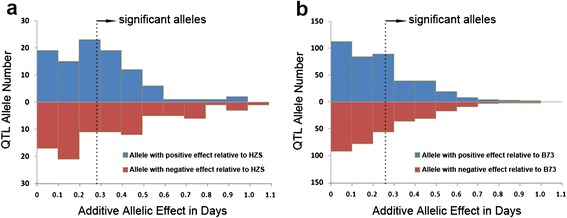
QTL allele effect size distributions for days to tasseling (DT) within the two maize NAM populations. **a** Additive allele estimates for the 18 DT QTLs for all 11 diverse parents relative to common parent HZS in CN-NAM. A total of 91 QTL alleles were significant at *P* < 0.05. **b** Additive allele estimates for the 29 DT QTLs for all 25 diverse parents relative to common parent B73 in US-NAM. A total of 232 QTL alleles were significant at *P* < 0.05

**Fig. 4:**
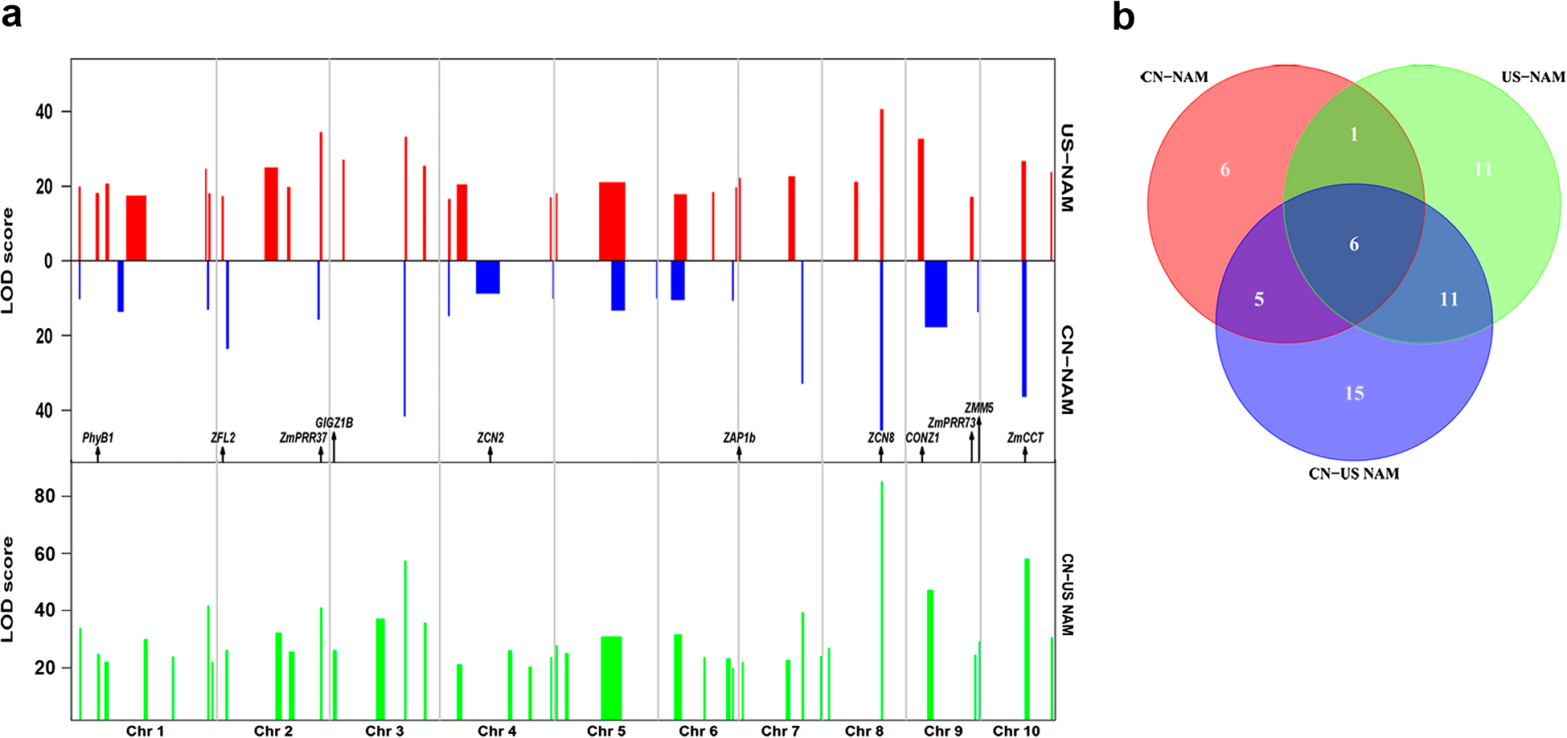
Comparison of QTL mapping results for days to tasseling (DT) among the US-NAM, CN-NAM, and CN-US NAM populations. **a** QTL mapping results for DT. The physical distance for each chromosome is represented in Mb (Mega-base) units on the horizontal axis. Bar width represents QTL confidence interval. Arrows represent the physical positions of the known maize flowering-time genes overlapping with QTL. **b** Venn diagram showing numbers of unique and shared QTLs among US-NAM, CN-NAM, and CN-US NAM

For QTL mapping of single NAM populations, a total of 18 and 29 QTLs were detected for DT in CN-NAM and US-NAM, respectively (Additional file 6: Table S5). About 46 % and 32 % of the DT-related QTL alleles had a significant effect at *P* < 0.05 for CN-NAM and US-NAM, respectively (Fig. 3); however, only 6.5 % (CN-NAM) and 1.7 % (US-NAM) had an additive effect on DT of about 1 day. About 17 % of the DT-related QTLs in CN-NAM and 28 % in US-NAM completely overlapped with the physical position of cloned genes controlling maize flowering time (Fig. 4a). Two well-studied maize flowering-time genes, *ZCN8* and *ZmCCT,* were located in QTL regions for both NAM populations.

### Joint linkage analysis for combining CN and US NAM populations

A composite genetic map for the combined CN-US NAM population was constructed based on 6,238 recombination bins, with a genetic length of 1,455.48 cM and an average genetic distance of about 0.23 cM between adjacent bins (Additional file 8: Table S6). When QTL mapping was conducted for DT in the CN-US NAM population, 37 QTLs were detected, which was significantly more than the total QTL number identified in a single NAM population (Fig. 4a). A comparative analysis revealed that six QTLs were shared among all three populations (US-NAM, CN-NAM, and CN-US NAM). Seventeen QTLs were shared between CN-US NAM and US-NAM, and 11 QTLs were shared between CN-US NAM and CN-NAM (Fig. 4b). Eight of 15 unique QTLs in CN-US NAM could be found in the single-family QTL mapping for both NAM populations (Additional file 5: Table S4).

The average QTL confidence intervals (CI) were 10.5 Mb and 9.3 Mb in CN-NAM and US-NAM, respectively. The average CI in CN-US NAM was 5.3 Mb; thus, combining populations improved the resolution by about 50 %. The QTL with the largest effect on DT had its LOD peak in the region that ranged from 123.810 Mb to 124.356 Mb on chromosome 8, while a gene *ZCN8* was about 300 kb apart from the peak. The QTL with the second largest effect on DT had its LOD peak on chromosome 10, between 94.588 Mb and 94.964 Mb, while a strong candidate gene *ZmCCT* was about 340 kb apart from the peak. Therefore, joint analysis for the combined CN-US NAM population was effective and valuable for quantitative trait mapping.

### Power and resolution of QTL mapping using different marker densities

**Fig. 5:**
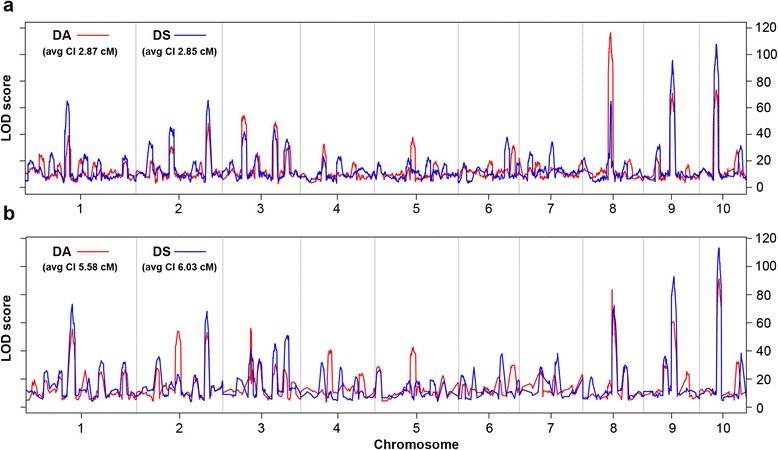
QTL mapping for days to anthesis (DA) and days to silking (DS) with different marker density maps in US-NAM. **a** Composite genetic map constructed using 4,932 bins. **b** Composite genetic map consisting of 1,106 SNPs published by McMullen et al. [43]. Avg CI in (**a**) and (**b**) represented average QTL confidence interval for DA and DS, respectively

The US-NAM population has been phenotyped for days to anthesis (DA) and days to silking (DS) in six environments in the US, and a medium-density composite genetic map was constructed using 1,106 SNPs [9]. Using the high-density composite genetic map of 5,296 bins, we reanalyzed DA and DS using the same phenotype data and mapping method. The number of QTLs and the total phenotypic variation explained by all QTLs were consistent with the results obtained using the medium-density map (Fig. 5). However, with the high-density map, the QTL CI varied from 1.0 to 6.4 cM and 0.4 to 7.8 cM, for DA and DS, respectively, with an average CI of 2.86 cM in both traits; whereas, using the medium-density map, the average QTL CI values were 5.58 and 6.03 cM for DA and DS, respectively. Two major QTLs controlling flowering time, *vgt1* and *ZmCCT*, had the most significant effects in US-NAM. The *vgt1* locus could be narrowed down from a 5-Mb region on the medium-density map to a 1.9-Mb region on the high-density map. *ZmCCT* was located in a 12.4-Mb region on the medium-density map, but could be narrowed to a 6.3-Mb region using the high-density map. These results suggest that recombination bin maps could be effectively used in fine mapping of genes/QTLs that control traits of interest.

## Discussion

GBS is an efficient and economical method, especially for genotyping RIL populations in which regions of extended linkage disequilibrium (LD) are common. We used the GBS method to genotype the US-NAM and CN-NAM populations and construct the genetic recombination maps for each family within the populations. Compared with genetic maps constructed using array-based SNP genotyping for the same populations [9, 33], marker numbers significantly increased, reducing the average marker interval. We found substantial biological variation in genetic map lengths per family within the two NAM populations.

The largest genetic map lengths in the 36 families were likely impacted by residual heterozygosity in the parents. In contrast, the smallest genetic map lengths were in families having large identity by descent (IBD) regions with a recurrent parent, for example, the K12 and HUANGYESI3 families. The marker density of the composite genetic maps for CN-US NAM was also four times higher than that published for US-NAM. The composite genetic map length in US-NAM was shorter than in CN-NAM, which may have resulted from the higher genotype calling error rate in CN-NAM due to higher residual heterozygosity.

We noticed that the number of bins generated from our study is slightly different from that of the previous study by Giraud et al. [34]. In addition to the different populations used, other causes might include different types of genetic markers and the methods used for the analyses. As the GBS markers in our study have a higher missing rate and genotyping error potentially, we are on the conservative side for calling bins. The benefit is the effective reduction of genetic markers for joint linkage analysis by using stepwise regression.

First, we filtered out SNPs with low quality by using permutations involving resampling of windows of SNPs followed by Bayesian inference [35]. Then consecutive SNPs with the same genotype across all RILs were lumped into blocks, and a recombination breakpoint was assumed at the transition between two different homozygous genotype blocks. The genotypic maps of the RILs were aligned and split into recombination bins [26]. Finally, after obtaining a skeleton recombination bin map, we further reduced the number of bins by merging bins less than 5 kb apart. Consequently, our results do not suggest any reduction of false double recombinant in our analyses.

The quality and accuracy of the genetic maps in this study were confirmed by QTL mapping of flowering-time-related traits. The co-location of major QTLs and cloned genes controlling flowering time in maize provided evidence that high-resolution genetic maps are possible using the bin mapping strategy. QTL mapping of CN-NAM and US-NAM identified 18 and 29 significant DT-related QTLs, respectively. Of these identified QTLs, 17 % of the CN-NAM ones and 28 % of those in US-NAM overlapped with previously cloned flowering-related genes in maize. These results illustrate the potential power and reliability of bin-based, composite genetic map construction. Hence, we strongly suggest that this method could become an efficient, accurate, and low-cost approach for primary or fine QTL mapping in maize, and other species.

The US-NAM analysis of maize flowering-time traits argued for numerous small-effect QTLs with additive effects [9]. We wondered if this conclusion was the product of the way the parents were selected or was indicative of more general phenomena. To directly evaluate this question, we contrasted the US-NAM analysis with a parallel analysis of CN-NAM. The common parents of the two NAM populations were chosen based on a similar strategy. Both HZS in China and B73 in the US are very important and widely deployed elite inbred lines in each country’s history of maize breeding. Diverse parents of the two NAM populations were derived from different germplasm sets. Previously, numerous small-effect QTLs were identified for flowering time in US-NAM, phenotyped for flowering time in six different US environments [9]. Likewise, flowering time evaluation of US-NAM, collected from different Chinese environments, showed no evidence for any single large-effect QTL. Although we found a higher percentage of significant QTL alleles with additive DT effect in CN-NAM (46 %) compared to US-NAM (32 %), no large additive effect was evident. Therefore, CN-NAM results supported the earlier US-NAM finding that flowering-time traits of NAM populations were controlled by numerous small-effect QTLs with additive effects. Additionally, we found a higher percentage of alleles with additive DT effects of about 1 day in CN-NAM (6.5 %) compared to US-NAM (1.7 %). This result may be due to the bigger difference in flowering times between the common parent and diverse parents in CN-NAM compared to those in US-NAM.

The power to detect QTL with a high-density map did not improve compared to that using a medium-density map. This is in agreement with results from Stange et al. [36] showing that high-density maps could not improve QTL detection power in experimental and simulation data with marker densities of 1, 2, and 5 cM. Factors influencing the statistical power of QTL mapping include mapping population size, marker density, QTL effect size, and significance level in declaring the existence of QTL. Previous studies suggested that the power of QTL detection was little affected by an increase in marker density beyond 10 cM, irrespective of population size and size of QTL effects [37, 38]. In our study, the medium-density map had a marker density of 1.30 cM. Hence, higher marker densities provide no advantage on the increase of the QTL detection power.

However, we did find a considerable improvement in mapping resolution using a high-density map compared to a medium-density map. For example, the average lengths of confidence intervals for DA- and DS-related QTLs were narrowed by 60 % and 47 %, respectively, using the high-density map. This is consistent with results reported by Stange et al. [36], where the QTL confidence interval lengths decreased with an increase of marker density regardless of population size and QTL effect size. High-density maps are beneficial for narrowing LOD peaks of QTLs and improving the precision of QTL localization, since they could increase the probability that a marker is tightly linked to a QTL. In our study, this improved resolution could be beneficial for fine mapping of QTLs and for marker-assisted breeding. The two NAM populations with high-density marker maps can be used to map QTLs for other quantitative traits, improving the power and resolution of QTL mapping. The QTL results detected in these two NAM populations can be mutually verified and QTL regions can be further narrowed by determining overlaps with QTLs detected in other maize NAM populations. Accumulation of results by expanding the use of this methodology in other NAM populations can provide an excellent platform for the dissection of the genetic architecture of complex agronomic traits in maize.

In this study, we estimated the confidence intervals of QTLs by using a 2-LOD drop-off method, which is widely used to investigate the QTL mapping resolution [34, 39, 40]. Although there is potential bias caused by stepwise regression, the chances have not been found that the bias would switch the order for comparing different procedures or methods. This suggests that our conclusion is very likely true in comparing the mapping resolutions by using a single NAM population or joint NAM populations.

The high density of molecular markers is one important limiting factor for generating high-resolution genetic maps. With ongoing advances in next-generation sequencing, marker density will continue to increase. However, ultra-high density markers will provide additional challenges for constructing genetic maps and conducting joint linkage analyses. Choosing representative SNPs from raw data is one way to produce a genetic map, but other important SNP information may be lost. Construction of bin maps uses all marker information to accurately evaluate the location of recombination breakpoints. Especially in NAM populations, we illustrated that this bin map method effectively reduced the computing time of QTL mapping without reducing mapping resolution.

This paper reports that the combination of CN and US NAM populations was fruitful for detecting the genetic architecture of complex traits in maize. Thus, this method has the potential to pave the way toward fine mapping of complex traits. Two additional NAM populations have been created within the European CornFed project [34]. In the future, the combination of more NAM populations with broader genetic diversity can benefit the systematic investigation of genetic architectures of complex traits in maize. In particular, NAM populations established based on European germplasm may be a good choice for integration with temperate maize. In contrast, NAM populations based on tropical and sub-tropical germplasm may be valuable for investigating important traits such as adaptation and abiotic stress tolerance.

## Conclusion

In this study, high-density and high-quality composite genetic recombination maps, based on genomic sequencing data, were generated for two NAM maize populations, US-NAM and CN-NAM. Using these maps, we were able to replicate the identification of previously known genes that affect flowering time in maize and improve the resolution of QTL mapping. The combined analysis of different NAM populations could improve the power and resolution of QTL detection compared to single NAM population mapping. This paper presents the necessary bin maps and computational methods for the maize scientific community to use these populations in their own research.

## Methods

### Plant material

The nested association mapping population from China (CN-NAM) is composed of about 2,000 RILs derived from crosses between the common parent HUANGZAOSI (HZS) with each of 11 diverse inbred lines: K12, YE478, ZHENG58, HUOBAI, QI319, WEIFENG322, LV28, HUANGYESI3, DUO229, PA405, and MO17. These are elite inbred lines and represent members of several popular heterotic groups used in Chinese maize breeding. HZS is an elite foundation parent in China, with wide adaptability, high combining ability, moderate growth period, and resistance to northern leaf blight and dwarf mosaic virus [19]. HZS has at least 70 derived lines and 80 hybrids. The K12 and HUANGYESI3 inbred lines were the derived lines of HZS [41]. HZS was crossed as the male parent to the other 11 inbred lines, and then an average of 180 RIL lines per family were derived through single seed descent (SSD) to the F_7_ generation. These RIL lines are free for public research purposes [42].

The US-NAM population, described in detail by McMullen et al. [43], consists of about 5,000 RILs derived from crossing B73 with 25 diverse inbred lines.

### Genotyping by sequencing

For the CN-NAM and US-NAM populations, a total of 7,698 RILs, including 758 repeatedly sequenced lines, were genotyped using the genotyping-by-sequencing (GBS) method [20]. A detailed protocol is described on the website given in Ref. [44]. US-NAM GBS data have been posted on the Panzea website [45]. CN-NAM GBS data are available from [42] and [45].

### Identification of outliers

Outliers were defined as containing non-parental alleles or retaining excess heterozygosity. Outliers were identified using unimputed GBS data of the NAM populations via the software TASSLE4.0 [46]. After filtering SNP sites (taxa coverage >10 %, minor allele frequency (MAF) >0.01, and site coverage >20 %), a neighbor-joining tree of every chromosome was made to spot the contaminated lines. If a line was not included in the corresponding family, the line was considered a contaminant. Lines with excess heterozygosity were identified for each family after filtering the data (site coverage >66 % and MAF >0.25). A line with a heterozygosity ratio >10 % was treated as an excess heterozygosity outlier.

### Bin map construction

SNP sites with MAF <0.05 and within the same tag (64 bp) based on the unimputed GBS data were filtered out for each family. The draft parental genotypes were inferred from the low-coverage SNP datasets of each RIL family using a maximum parsimonious inference of recombination (MPR) method applied in an R package MPR [35]. Then, parental genotypes were refined after removing low-quality SNPs by resampling and using the Bayesian inference method included in the MPR package. The genotype assignment of each RIL was performed using a hidden Markov model (HMM) approach, with heterozygote set to missing according to the method described by Xie et al. [35]. Consecutive SNP sites with the same genotype were lumped into blocks, and a breakpoint was assumed at the transition between two different genotype blocks. Blocks with lengths less than 1,500 kb and with a number of sequenced SNPs fewer than five were masked as missing data to avoid false double recombination. Markers co-segregating in two contiguous blocks were combined into a recombination bin [26]. After merging a bin smaller than 5 kb to the next bin, a skeleton bin map of RIL population was obtained. Genotypes of bins for regions at the transitions between two different genotype blocks were set to missing data and imputed using the R/qtl package [47]. The genetic maps for individual CN-NAM and US-NAM families were constructed from bins serving as genetic markers using the R/qtl package function *est.map* with the Haldane mapping method [47].

After inferring parental genotypes and obtaining high-quality SNPs for each family, markers polymorphic in more than two families in the CN-NAM population and four families in the US-NAM population were chosen to build composite genetic maps. Markers were encoded by designating the common parent allele as “1”, the other 36 parent alleles as “0”, and heterozygous loci as missing data “NA”. Markers monomorphic in a particular family were also converted to missing data “NA”. Joint recombination bin maps were generated by combining consecutive SNP sites with the same genotype into bins and merging bins smaller than 5 kb to the next bin using MPR (R package). After obtaining recombination bins, two composite genetic maps were constructed by using bins as markers in the JoinMap 4.0 software [48]. Markers were assigned into linkage groups at an independence test LOD score of 10. Due to the large number of markers in the two NAM populations, we used the maximum likelihood mapping algorithm to order loci. The Haldane mapping function was used to convert recombination frequencies to cM. For each linkage group in the two NAM populations, a framework reference map containing markers not exhibiting segregation distortion was constructed. Markers which significantly (*P* < 0.05) deviated from the expected 1:1 ratio in a chi-square test were defined as exhibiting segregation distortion. After the order and chromosome position of these markers were determined, the remaining markers with segregation distortion were added to the reference map. Any markers that showed inconsistent physical position in the RefGen_v2 with themselves were excluded from further mapping. The final selected map exhibited the most agreement in marker order with the framework map.

### Phenotyping

The 1972 CN-NAM lines were evaluated phenotypically in two years (2009 and 2010) and in three locations, i.e., Xinxiang in Henan province (35.19°N, 113.53°E), Beijing (39.48°N, 116.28°E), and Urumqi in Xinjiang province (43.47°N, 87.39°E). These locations represented three main maize growing regions in China. Each year/location combination was considered as an environment, generating a total of six environments. The CN-NAM population was summer sown in Henan and spring sown in Beijing and Xinjiang. Within each environment, trials included 11 separate sets (11 CN-NAM families), and each set contained all lines of one family and its two parents. For each set, all lines were randomly assigned within each replication with a one-row plot. Two replications of each set were planted adjacently. Each row included 11 plants and was 3 m long and 0.6 m apart. A set of 4,396 US-NAM lines were grown at five different environments in 2010 and 2011, i.e., Sanya in Hainan province (18.15°N, 109.30°E) in the winter of 2010, Xinxiang in Henan province (35.19°N, 113.53°E) in the summer of 2011, Beijing (39.48°N, 116.28°E) in the spring of 2011, Tianjin (39.10°N, 117.10°E) in the spring of 2011, and Chongqing (29.35°N, 106.33°E) in the spring of 2011. All trials were arranged in an augmented incomplete block design and consisted of one replication of US-NAM RILs and check entries [9]. For each location, lines were grouped by family with augmented incomplete blocks within each family. Each incomplete block comprised 50 RILs and one check: B73. Experimental units consisted of single-row plots of 11 plants. Each plot was 3 m in length with 0.6 m between rows. Days to tasseling (DT) data were collected for the CN-NAM and US-NAM populations and measured as the number of days from planting to tassel emergence for half the plants within a row or plot. The DT datasets for both NAM populations are available in Additional file 9: Table S7 and Additional file 10: Table S8. The broad-sense heritability for DT across the environments in different NAM populations was calculated on a mean basis by PROC GLM in SAS v9.2. The best linear unbiased prediction (BLUP) value of DT across environments was obtained for each line of CN-NAM and US-NAM with the MIXED procedure in SAS (SAS Institute Inc.). Subsequently, the BLUP value for both NAM populations was used for QTL mapping.

### QTL analysis

The method of inclusive composite interval mapping (ICIM) was used to detect the additive QTL for each of the 36 families by using the QTL ICI Mapping software Ver 3.2 [49]. In ICIM, the *P* values for entering variables (PIN) and removing variables (POUT) were set at 0.001 and 0.002, and the scanning step was set 1.0 cM. The LOD threshold was determined by a 1,000-permutation test.

Joint QTL mapping for a single NAM population or the two NAM populations together was conducted by using the stepwise regression model described by Buckler et al. [9]. We employed PROC GLMSelect in SAS v9.2 to conduct this analysis. In the stepwise regression model for a single NAM, the BLUP of days to tasseling (DT) was used as the response variable. The family main effects and 4,932 bin markers in CN-NAM or 5,296 bin markers in US-NAM were fitted as the explanatory variables. The family main effects were always included in the stepwise regression model. Then, marker effects nested within families were chosen to enter or leave the model with a threshold of the *P* value. The threshold was determined by permutation tests (1,000 times) for a corresponding type I error rate of 5 % [9]. For CN-NAM and US-NAM, the *P* values corresponding to a 5 % type I error were both approximately 0.0001.

In the stepwise regression model for both NAM populations, the BLUP of DT was used as the response variable. The family main effects, reference (B73 and HZS) effects, and 6,238 bin markers were the explanatory variables. The family effects and reference effects were fit first in the model. Then, marker effects nested within families were selected to enter or stay in the model based on the same procedure described above. In this situation, the *P* value threshold is *P* = 0.0005 for a corresponding type I error of 5 %. For each QTL selected from the stepwise regression model, the adjacent markers, four from each side, were tested to derive the LOD score, one at a time, with all the QTLs as covariates. For each QTL position identified, confidence intervals were constructed by a 2-LOD drop-off method.

Allelic effects of markers within a family were estimated from the final optimized QTL model. Significances of allele effects were determined with a *t*-test on the differences between their founder means and the reference (B73 or HZS) allele effect at a significance level of *P* = 0.05.

## Additional files

Additional file 1: Table S1. Summary of population size, recombination event number, and genetic map information within the 36 RIL families of the two NAM populations. (XLSX 10 kb) Additional file 2: Table S2. Recombination bin map information within the 36 families of the two NAM populations, based on high-quality SNPs obtained from population sequencing. (XLSX 4828 kb) Additional file 3: Figure S1. Joint recombination bin maps in (a) CN-NAM and (b) US-NAM. (a) CN-NAM map included 5,435 recombination bins for the 1,696 RILs. (b) US-NAM map included 5,692 recombination bins for the 4,623 RILs. Chromosomes are separated by vertical gray lines. X-axis represents the physical location of recombination bins in B73 RefGen_v2. Y-axis represents RILs in different families. (TIFF 6180 kb) Additional file 4: Table S3. Joint recombination bin map information for CN-NAM and US-NAM, respectively. (XLSX 638 kb) Additional file 5: Table S4. QTLs identified for days to tasseling trait within the 36 families of the two NAM populations. (XLSX 20 kb) Additional file 6: Table S5. Joint linkage analysis summary for days to tasseling trait in CN-NAM and US-NAM, respectively. (XLSX 14 kb) Additional file 7: Figure S2. Physical locations of days to tasseling QTLs and candidate genes in the 36 families. Chromosomes are separated by gray solid lines. Solid circle sizes represent different LOD values. Purple circles represent QTL allele effects from 36 diverse parents, green circles from common parents. Gray dotted lines represent the physical locations of candidate genes. (TIFF 3208 kb) Additional file 8: Table S6. Information about joint recombination bin maps for the combined CN-NAM and US-NAM populations. (XLSX 411 kb) Additional file 9: Table S7. Phenotypic data for days to tasseling in six environments for the CN-NAM population. (XLSX 193 kb) Additional file 10: Table S8. Phenotypic data for days to tasseling in five environments for the US-NAM population. (XLSX 179 kb)
