# Supplementary figures and images for: Construction of high-quality recombination maps with low-coverage genomic sequencing for joint linkage analysis in maize
Source: BMC Biol. 2015 Sep 21;13:78. doi: 10.1186/s12915-015-0187-4 (PMC4578237; doi:10.1186/s12915-015-0187-4)

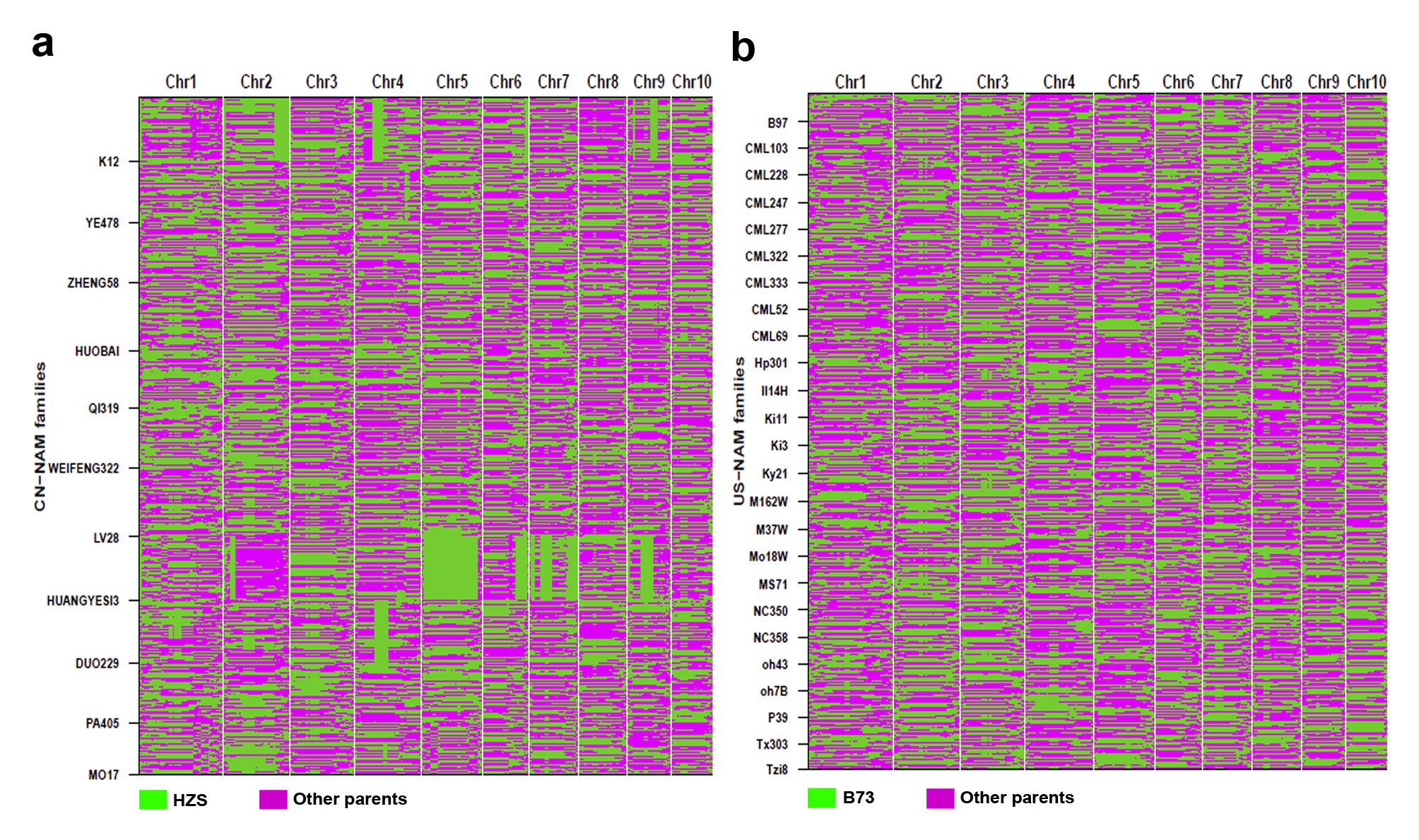

Supplement: Additional file 3: Figure S1. — Joint recombination bin maps in (a) CN-NAM and (b) US-NAM. (a) CN-NAM map included 5,435 recombination bins for the 1,696 RILs. (b) US-NAM map included 5,692 recombination bins for the 4,623 RILs. Chromosomes are separated by vertical gray lines. X-axis represents the physical location of recombination bins in B73 RefGen_v2. Y-axis represents RILs in different families. (TIFF 6180 kb) [file 12915_2015_187_MOESM3_ESM.tiff]

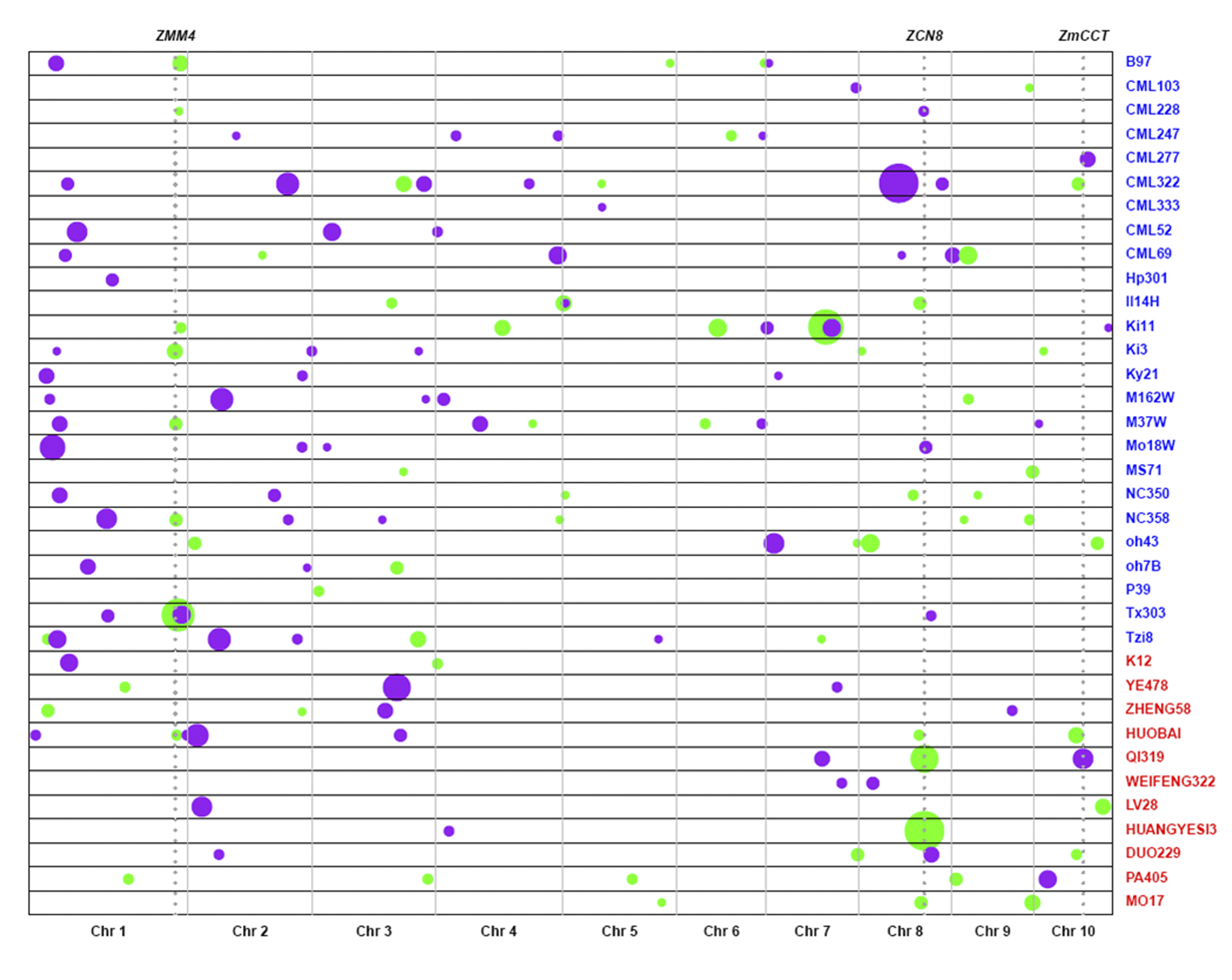

Supplement: Additional file 7: Figure S2. — Physical locations of days to tasseling QTLs and candidate genes in the 36 families. Chromosomes are separated by gray solid lines. Solid circle sizes represent different LOD values. Purple circles represent QTL allele effects from 36 diverse parents, green circles from common parents. Gray dotted lines represent the physical locations of candidate genes. (TIFF 3208 kb) [file 12915_2015_187_MOESM7_ESM.tiff]
